# Proteomic characterization of primary and metastatic prostate cancer reveals reduced proteinase activity in aggressive tumors

**DOI:** 10.1038/s41598-021-98410-0

**Published:** 2021-09-23

**Authors:** Qing Kay Li, Jing Chen, Yingwei Hu, Naseruddin Höti, Tung-Shing Mamie Lih, Stefani N. Thomas, Li Chen, Sujayita Roy, Alan Meeker, Punit Shah, Lijun Chen, G. Steven Bova, Bai Zhang, Hui Zhang

**Affiliations:** 1 Department of Pathology, The John Hopkins Medical Institutions, 600 N. Wolfe Street, Baltimore, MD 21224 USA; 2 Department of Oncology, Sidney Kimmel Cancer Center, Johns Hopkins Medical Institutions, Baltimore, MD USA; 3 Department of Urology, Sidney Kimmel Cancer Center, Johns Hopkins Medical Institutions, Baltimore, MD USA; 4 Prostate Cancer Research Center, Faculty of Medicine and Health Technology, Tampere University, FI-33014 Tampere, Finland; 5 Johns Hopkins University, 400 N. Broadway, Smith Bldg Rm 4011, Baltimore, MD 21287 USA

**Keywords:** Cancer, Chemical biology, Molecular biology, Biomarkers, Oncology, Urology

## Abstract

Prostate cancer (PCa) is a heterogeneous group of tumors with variable clinical courses. In order to improve patient outcomes, it is critical to clinically separate aggressive PCa (AG) from non-aggressive PCa (NAG). Although recent genomic studies have identified a spectrum of molecular abnormalities associated with aggressive PCa, it is still challenging to separate AG from NAG. To better understand the functional consequences of PCa progression and the unique features of the AG subtype, we studied the proteomic signatures of primary AG, NAG and metastatic PCa. 39 PCa and 10 benign prostate controls in a discovery cohort and 57 PCa in a validation cohort were analyzed using a data-independent acquisition (DIA) SWATH–MS platform. Proteins with the highest variances (top 500 proteins) were annotated for the pathway enrichment analysis. Functional analysis of differentially expressed proteins in NAG and AG was performed. Data was further validated using a validation cohort; and was also compared with a TCGA mRNA expression dataset and confirmed by immunohistochemistry (IHC) using PCa tissue microarray (TMA). 4,415 proteins were identified in the tumor and benign control tissues, including 158 up-regulated and 116 down-regulated proteins in AG tumors. A functional analysis of tumor-associated proteins revealed reduced expressions of several proteinases, including dipeptidyl peptidase 4 (DPP4), carboxypeptidase E (CPE) and prostate specific antigen (KLK3) in AG and metastatic PCa. A targeted analysis further identified that the reduced expression of DPP4 was associated with the accumulation of DPP4 substrates and the reduced ratio of DPP4 cleaved peptide to intact substrate peptide. Findings were further validated using an independently-collected tumor cohort, correlated with a TCGA mRNA dataset, and confirmed by immunohistochemical stains of PCa tumor microarray (TMA). Our study is the first large-scale proteomics analysis of PCa tissue using a DIA SWATH-MS platform. It provides not only an interrogative proteomic signature of PCa subtypes, but also indicates the critical roles played by certain proteinases during tumor progression. The spectrum map and protein profile generated in the study can be used to investigate potential biological mechanisms involved in PCa and for the development of a clinical assay to distinguish aggressive from indolent PCa.

## Introduction

Prostate cancer (PCa) is the most common cancer and the second leading cause of cancer death in men in the United States, with an estimated 192,000 new cases and 33,000 deaths in 2020^1^. The clinical behavior of PCa is highly variable. The majority of PCa cases present as localized and/or slow-growing disease, which can be safely observed by active surveillance without the need for invasive treatments. However, a subset of tumors reveals an aggressive behavior resulting in progression, metastasis and death^2^. Several recent studies have also demonstrated that the incidence rate for localized disease continues to decline, whereas; the incidence rate for advanced-stage disease continues to rise in men over 50 years of age^2–4^. This trend was also demonstrated by the study from the US Preventive Services Task Force (USPSTF)^5,6^. To separate the high-risk aggressive PCa (AG) from low-risk non-aggressive indolent tumors (NAG), multiple risk stratification systems have been developed, including the combination of both clinical and pathological parameters (such as Gleason score/ISUP grade, PSA levels, clinical and pathological staging); however, these tools are still fail to adequately predicting the disease progression and outcomes^7,8^.

Recently, further risk stratification using molecular features has been developed. The genomic analysis and the Cancer Genome Atlas (TCGA) demonstrate a substantial heterogeneity of PCa, including a spectrum of molecular abnormalities; and these findings are correlated with variable clinical courses PCa can take^9–14^. In the TCGA study^13^, the comprehensive genomic analysis of 333 primary PCa cases revealed that the majority of tumors (74%) fell into one of seven subtypes defined by specific gene fusions (*ERG*, *ETV1/4, FLI1*) or mutations (*SPOP*, *FOXA1*, *IDH1*). Furthermore, epigenetic profiles also found that PCa with the *IDH1*-mutation had a unique methylated phenotype^11–14^. In the *SPOP* and *FOXA1* mutants, the androgen receptor (AR) activity varied widely, having the highest levels of AR-induced transcripts. It was also reported that 25% of PCa had alterations in the PI3K or MAPK signaling pathways, and approximately 20% of PCa had abnormalities in DNA repair genes^9–14^. These studies reveal not only genomic heterogeneity to be among primary molecular aberrations in PCa, but also identify potentially actionable targets for therapeutic interventions.

The Sequential Window Acquisition of all theoretical fragment ion spectra (SWATH), also called data-independent acquisition (DIA) of mass spectrometry (MS), has emerged as an unbiased and alternative technology for proteomic analysis of biological samples that can overcome certain limitations of conventional data-dependent acquisition (DDA)-based analysis, such as the stochastic nature of precursor ion selection and low sampling efficiency^15–18^. Previous studies performed by us and others indicate that SWATH can create a comprehensive fragmentation map of all detectable precursors for accurate quantification of a given sample by dividing peptide precursor ions into several consecutive windows during fragmentation. Other benefits of the SWATH platform include requiring lesser quantity of clinical samples, providing sufficient proteome coverage with quantitative consistency, and analytic accuracy^15,19,20^. SWATH-MS is a fast, simple and reproducible method for large-scale quantitative proteomic analysis.

To further understand the molecular features of the PCa progression, we performed a proteomic analysis of primary (including both indolent NAG and AG subtypes) and metastatic PCa using the SWATH-MS platform. The purposes of our study were to generate a comprehensive proteomic map of AG, NAG and metastatic PCa by using a DIA approach and to identify unique AG-associated proteins, which might be used for the development of a clinical assay to separate AG from NAG PCa.

## Results

### Experimental design

Our cohort consisted of 106 fresh frozen prostate tissues, including 48 primary and 48 metastatic PCa and 10 benign prostate tissue controls (C) (Supplementary Table 1 ) In primary PCa, 29 cases were indolent NAG with a Gleason score of 6 (follow-up data up to 20 years); and 19 cases were AG with a Gleason score > 7 (died of PCa). In metastatic PCa, 38 cases were treated with androgen deprivation therapy (castration resistant metastases, M) and 10 cases were treated without androgen deprivation therapy (castration naïve prostate metastases, Nmet). Autopsies were also performed in deceased cases as part of the Project to Eliminate Lethal Prostate Cancer (PELICAN) and the Johns Hopkins Autopsy Study of Lethal Prostate Cancer (JHASPC). All benign prostate controls were collected from healthy transplant donors who died of conditions other than PCa. In our study, the AG and NAG were defined by the criteria of the International Society of Urological Pathology^5,6^. The median tumor purities were 70% ± 11%, 50% ± 17% and 70.0% ± 15% in metastatic PCa, NAG and AG, respectively.

**Figure 1:**
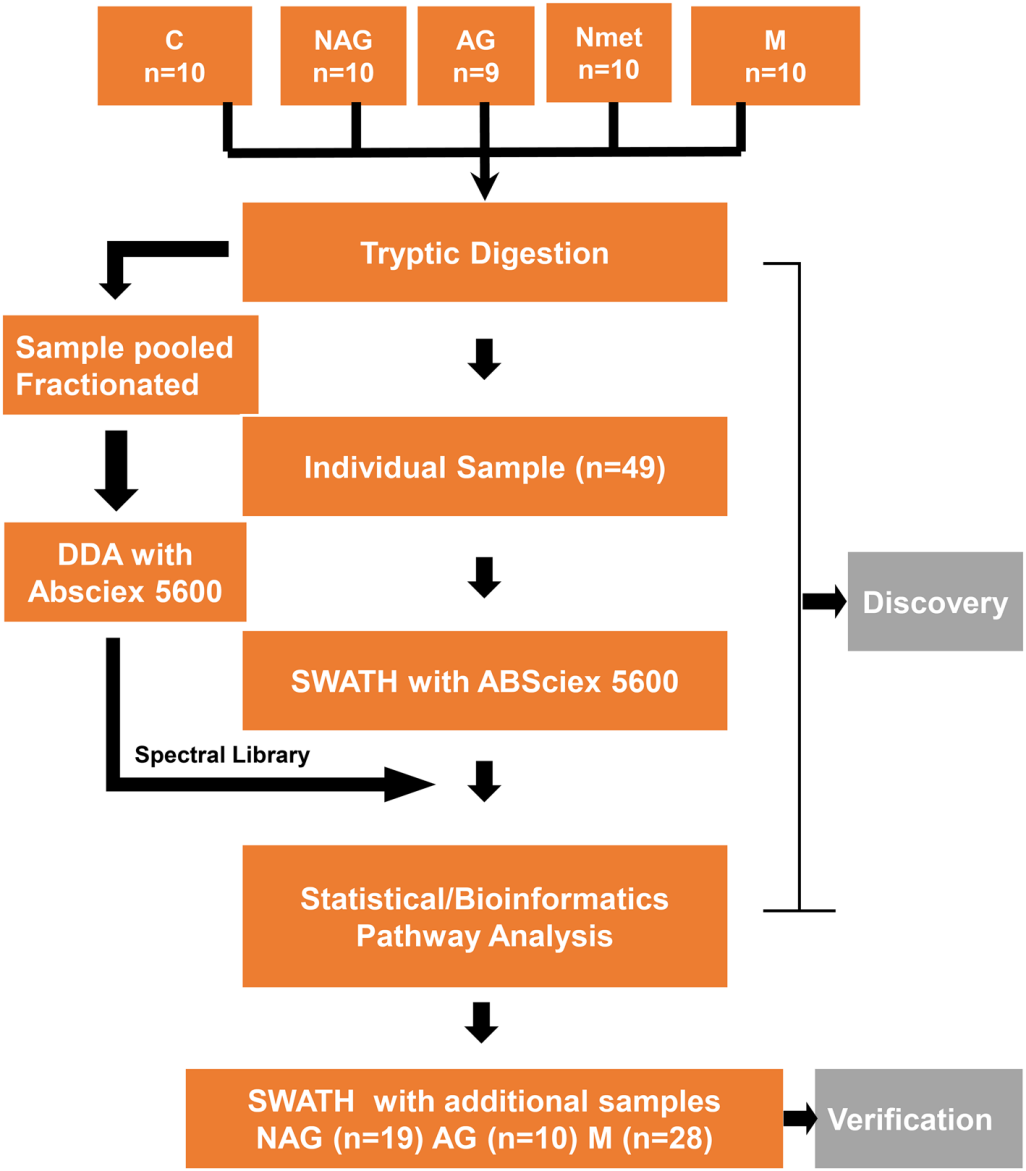
Schematic diagram of the workflow. First, we characterized the protein signatures of 49 cases in the discovery cohort, including 19 primary tumors (10 NAG and 9 AG), 20 metastatic tumors (10 Nmet and 10 M) and 10 benign controls (C). Next, we further validated our findings by the targeted re-examination of SWATH-MS maps using the independently collected cohort, including 29 primary PCa (19 NAG and 10 AG) and 28 metastatic tumors (M). C: benign control, NAG: non-aggressive PCa, AG: aggressive PCa, Nmet: metastasis without androgen deprivation therapy; M: metastasis with androgen deprivation therapy.

Proteomic profiles were generated using the SWATH-MS analytic platform. The workflow is demonstrated in Fig. 1. We first characterized the protein signatures of PCa in the discovery cohort, which consisted of 5 different groups (10 C, 10 NAG, 9 AG, 10 Nmet and 10 M). The SWATH data files from individual samples were searched against a customized spectral library constructed from a combined sample pool and analyzed using data dependent LC–MS/MS on 5600^+^ Triple TOF and the Human Proteome Map. We further validated our findings (verification phase) with a targeted re-examination of SWATH-MS maps using an independently collected cohort, including 19 NAG, 10 AG and 28 M.

### Global proteomic profile of non-aggressive, aggressive and metastatic PCa

The proteomic profiles of PCa and 10 benign controls were performed for comprehensive proteome characterization. Using the spectra from DDA runs, a total of 4,415 proteins were identified and quantified from SWATH maps, with an average protein identification number of 1275 ± 246 in an individual run (Supplementary Table 2). 198 proteins were consistently quantified across all samples and 761 proteins are quantified in at least 75% samples with FDR < 0.01. We also evaluated data reproducibility through the analysis. In the study, 48 samples were analyzed in duplicate. The reproducibility of the protein measurements was assessed using these replicated runs. A correlation score of 0.89 was achieved among replicates, indicating a satisfactory reproducibility of the SWATH-MS workflow in quantification of the proteome data.

**Figure 2:**
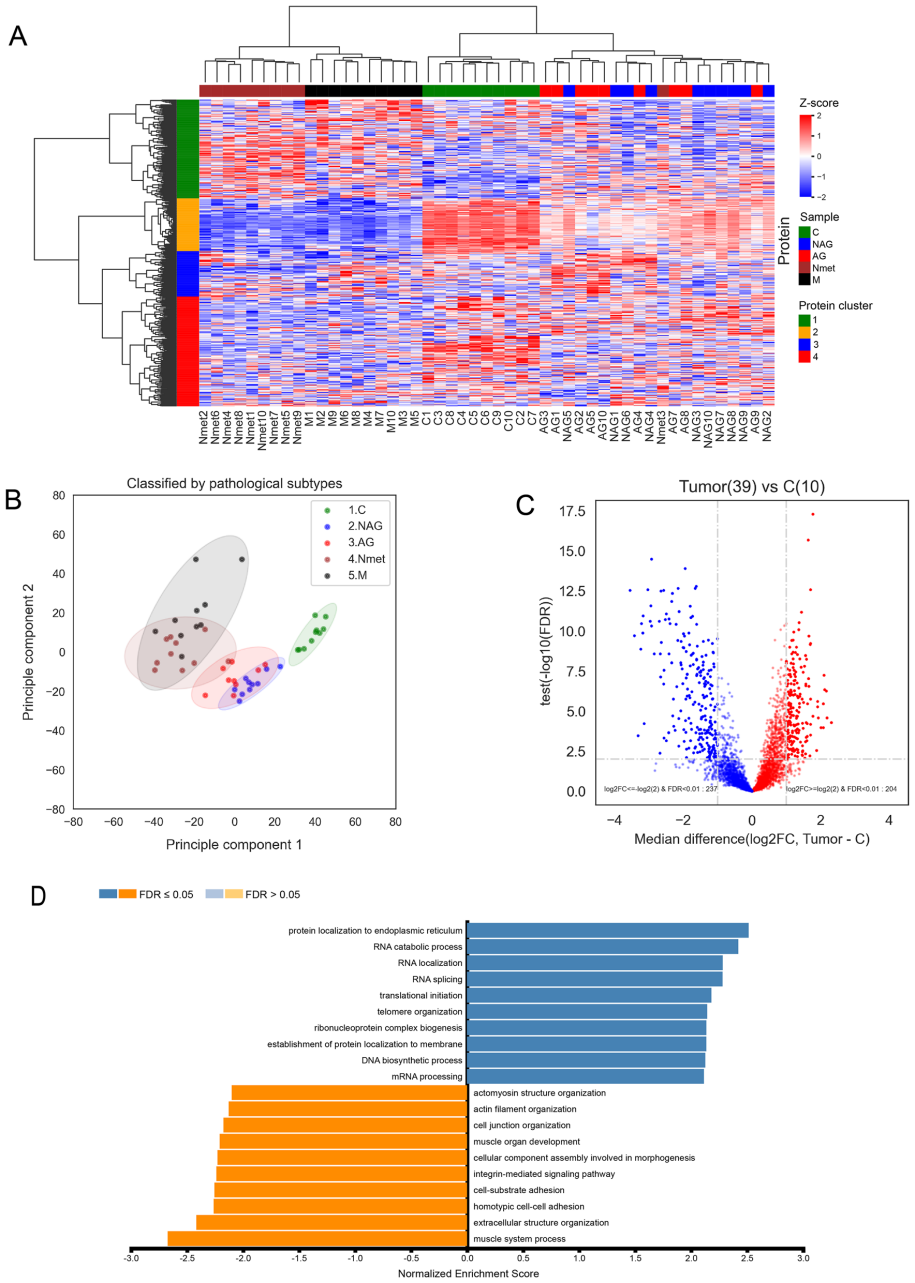
SWATH-MS analysis of PCa. (**A**) non-supervised hierarchical clustering of top 500 most variant proteins. Sample types were annotated by colors beyond the heat map as: NAG (blue), AG (red), Nmet (brown), M (black) and C (green). Protein clusters were labelled by colors on the left side of the heat map as: cluster 1 (green), cluster 2 (orange), cluster 3 (blue), and cluster 4 (red). (**B**) PCA analysis. (**C**) Volcano plot of significantly expressed proteins in tumors (NAG, AG, Nmet, and M groups) and controls C. (**D**) Gene set enrichment analysis (GSEA) of differentially expressed proteins based on biological process terms in the gene ontology database accessed via WebGestalt.

We investigated the protein differential expression patterns in all samples. The non-supervised hierarchical clustering was performed on the top 500 most variant proteins. The heat map was constructed using cancer-associated proteins. The expression values were transformed into Z scores at the protein level (Fig. 2A). Four major protein clusters were identified among PCa subtypes, including NAG, AG, metastases and benign control C. Furthermore, several sample clusters were also identified among individual samples. To further correlate the major protein clusters with individual samples, biological processes (BP) annotation in each major protein cluster was generated based on Over-Representation Analysis (ORA) using WebGestalt. Among the four major protein clusters, we found that the Gene Oncology (GO) terms of muscle contraction (cluster 2 in orange color), transcriptional dysregulation in cancer (cluster 3 in blue color) and immune response (cluster 4 in red color) were enriched (Fig. 2A, Supplementary Table 3). There was no significant GO term enriched in cluster 1 (green color). These biological annotations were correlated with individual sample clustering. For example, protein cluster 2 demonstrated a differential pattern between primary and metastatic PCa.

The Principal Component Analysis (PCA) of all samples further illustrated the differences of protein expression among different sample groups (Fig. 2B). In the analysis, the tumor subtypes demonstrated distinct patterns in comparison to the benign control. The benign control, primary tumor (NAG and AG), and metastatic tumor (Nmet and M) groups, except one case, could be separated based on their protein expression patterns, demonstrating distinctive protein patterns.

Through further comparison of tumors (NAG, AG, Nmet and M) with controls, we identified 204 significantly up-regulated and 237 down-regulated proteins (adjusted p-value < 0.01 and absolute fold change ≥ 2) (Fig. 2C, Supplementary Table 3). Based upon the Gene Set Enrichment Analysis (GSEA), we found different protein localizations within up- and down-regulated proteins. The top three BP terms enriched in up-regulated proteins were endoplasmic reticulum, RNA catabolic process, and RNA localization, whereas, the top three BP terms enriched in down-regulated proteins were muscle system process, extracellular structure organization, and homotypic cell–cell adhesion (Fig. 2D, Supplementary Table 3).

### Functional analysis revealed downregulation of proteinases

**Figure 3:**
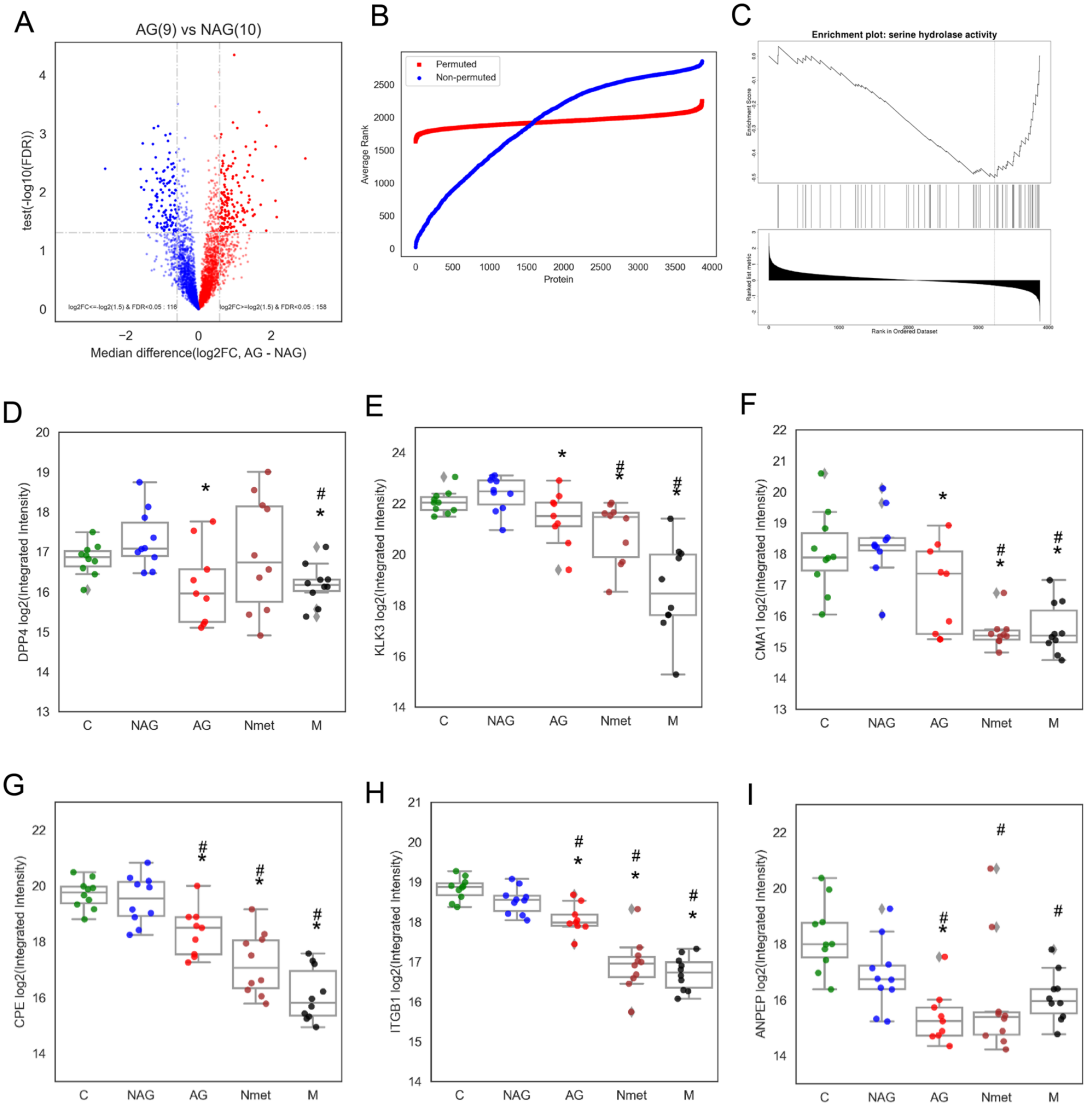
Functional analysis of different subtypes of PCa. (**A**) Volcano plot of differentially expressed proteins between NAG and AG groups. (**B**) Permutation test for the selection of stable proteins discriminating between NAG and AG groups. (**C**) Functional enrichment of proteinases suggested by GSEA analysis via WebGestalt. (**D**) Expression patterns of DPP4, (**E**) Expression patterns of KLK3, (F) Expression patterns of CMA1, (**G**) Expression patterns of CPE, (**H**) Expression patterns of ITGB1, and (**I**) Expression patterns of ANPEP in different tumor subtypes. *P < 0.05 (NAG vs other subtypes). #: *P* < 0.05 (C vs other subtypes).

We further analyzed the signature of differentially expressed proteins between NAG and AG groups. We identified 158 up-regulated and 116 down-regulated proteins with subtype-associated differential expression (*P*-value < 0.05 and fold change ≥ 1.5 (Fig. 3A, Supplementary Table 3). All the proteins with subtype-associated differential expression were further verified by the permutation test as stable proteins discriminating between AG and NAG primary tumors (Fig. 3B, Supplementary Table 4).

We performed the molecular function (MF) annotation using WebGestalt to categorize biological functions of AG tumor-associated proteins. The serine hydrolases activity was associated with a reduced expression pattern (Fig. 3C, Supplementary Table 4). The most differentially expressed proteinases were dipeptidyl peptidase 4 (DPP4), prostate specific antigen (KLK3), and chymase (CMA1) (Fig. 3D–F). Similar expressional patterns were also found in carboxypeptidase E (CPE), integrin beta-1 (ITGB1) and aminopeptidase N (ANPEP) (Fig. 3G–I). Our data demonstrated that these proteinases were markedly downregulated in primary AG and the majority of metastatic PCa.

### Further verification of proteinases signature using independent cohort and a transcriptomic dataset

**Figure 4:**
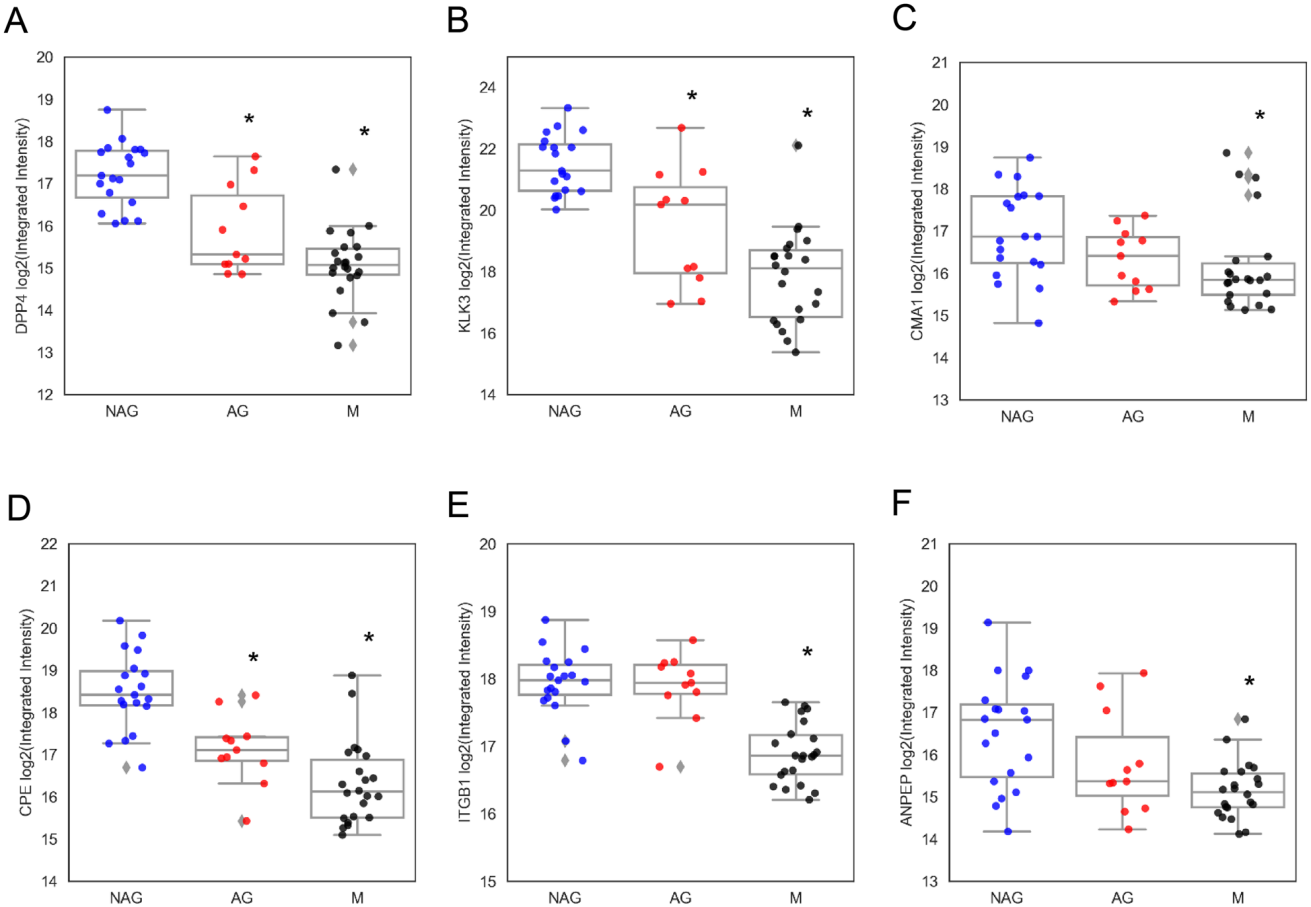
Verification of dysregulation of proteinases using independently collect cohort. Proteinase expression patterns of DPP4 (**A**), KLK3 (**B**), CAM1 (**C**), CPE (**D**), ITGB1 (**E**), and ANPEP (**F**) in different subtypes of tumors, including NAG, AG and metastatic PCa (M). *p < 0.05.

We further verified the altered proteinases expression of serine hydrolase in the discovery samples set using an independently collected cohort, containing 29 primary PCa and 28 metastatic tumors (Fig. 4). We found similar expression patterns of DPP4, KLK3 and CMA1 in the validation sample set (Fig. 4A–C). Proteinases CPE, ITGB1 and ANPEP also revealed similar expression patterns (Fig. 4D–F).

**Figure 5:**
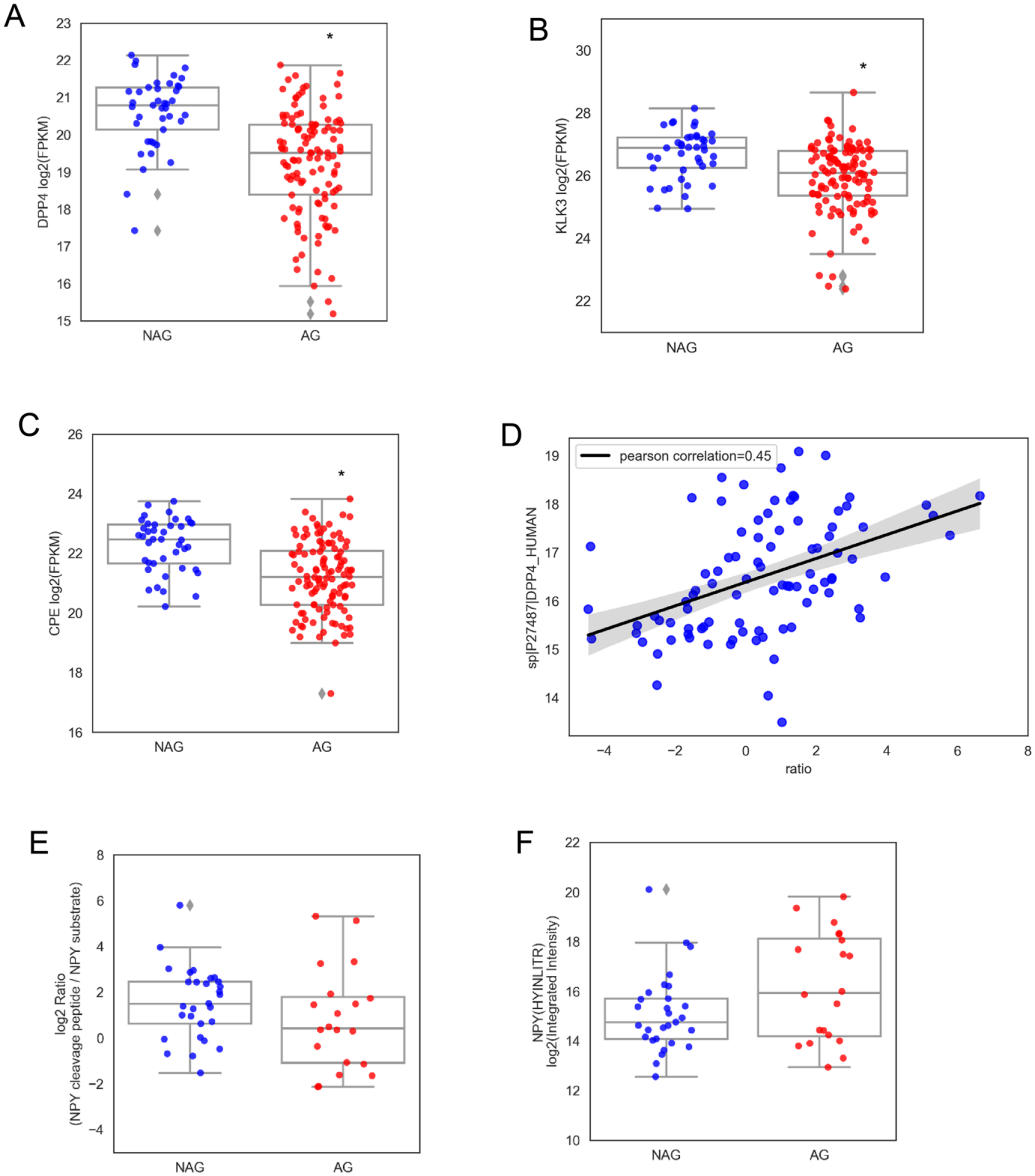
Loss of DPP4 transcription and activity in AG tumors. (**A**) DPP4 mRNA expression of AG tumors from TCGA dateset. (**B**) KLK3 mRNA expression of AG tumors from TCGA dataset. (**C**) CPE mRNA expression of AG tumors from TCGA dataset. (**D**) Correlation of DPP4 expression with the ratio of NPY cleaved peptide to intact substrate peptide. (**E**) The ratio of NPY cleaved peptide to intact substrate peptide in AG tumors. (**F**) Total NPY peptide expressions between NAG and AG tumors. *P < 0.05.

To assess whether the observed decreased expression of proteinases was regulated at the transcriptional level, we interrogated and compared our findings with the TCGA prostate adenocarcinoma RNA-seq dataset. The RNA-seq data (mRNA z-score) and clinical information from TCGA study was downloaded from the Genomic Data Commons Data Portal (https://portal.gdc.cancer.gov/). In this analysis, we only included TCGA PCa cases without history of any other malignancy and no neoadjuvant treatment. Among the TCGA cases, 114 were identified as AG tumors (Gleason score ≥ 7, nor biochemical recurrence, nor involvement of regional lymph nodes, nor clinical identified metastases); and 43 were identified as NAG tumors (Gleason score of 6, nor biochemical recurrence, nor lymph nodes involvement or clinical identified metastases) (Supplementary Table 5). The mRNA expressions of DPP4, CPE and KLK3 were markedly down-regulated in AG PCa in the TCGA dataset (Fig. 5A–C). The comparison of our proteomics data with TCGA data demonstrated that our findings of down-regulated expression of proteinases were in agreement with the mRNA expression pattern.

### Loss of DPP4 correlate with the accumulation of substrates in aggressive PCa

We further analyzed proteolytic product abundance in NAG and AG by re-examination of the SWATH data for substrate levels of DPP4. Two forms of DPP4 related products were identified, including the intact peptide neuropeptide Y (NPY(1–36)) containing the sequence of YPSKPDNPGEDAPAEDMAR, and the cleaved peptide of NPY (NPY (3–36)) containing the sequence of SKPDNPGEDAPAEDMAR, where 2 amino acids are truncated from the N-terminus of intact NPY(1–36) by DPP4 enzymatic activity ^21,22^. Both these two peptides contain c-terminal sequence of HYINLITRQR.

Based upon SWATH-MS data from all 106 cases of PCa and benign controls, we quantified 3 peptides including the intact NPY (1–36), cleaved peptide of NPY (3–36) and c-terminal peptide of NPY. A positive correlation between DPP4 protein expression and NPY cleaved peptide (SKPDNPGEDAPAEDMAR/*YP*SKPDNPGEDAPAEDMAR) was found with a Pearson correlation coefficient of 0.45 (Fig. 5D), indicating that peptidase activity of DPP4 was correlated with an accumulation of its substrate NPY. A lower ratio of NPY cleaved peptide to intact substrate peptide (SKPDNPGEDAPAEDMAR/*YP*SKPDNPGEDAPAEDMAR ratio) was observed in the AG tumors (Fig. 5E), further confirming a reduced DPP4 activity in aggressive tumors. The reduced cleaved peptide (NPY 3–36) and accumulation of intact substrate peptide (NPY 1–36) were also identified by the detection of peptides containing the c-terminal sequence of HYINLITRQR (Fig. 5F).

### Verification of reduced DPP4 expression as a signature of aggressiveness by PCa tissue microarray (TMA) and immunohistochemistry (IHC)

**Figure 6:**
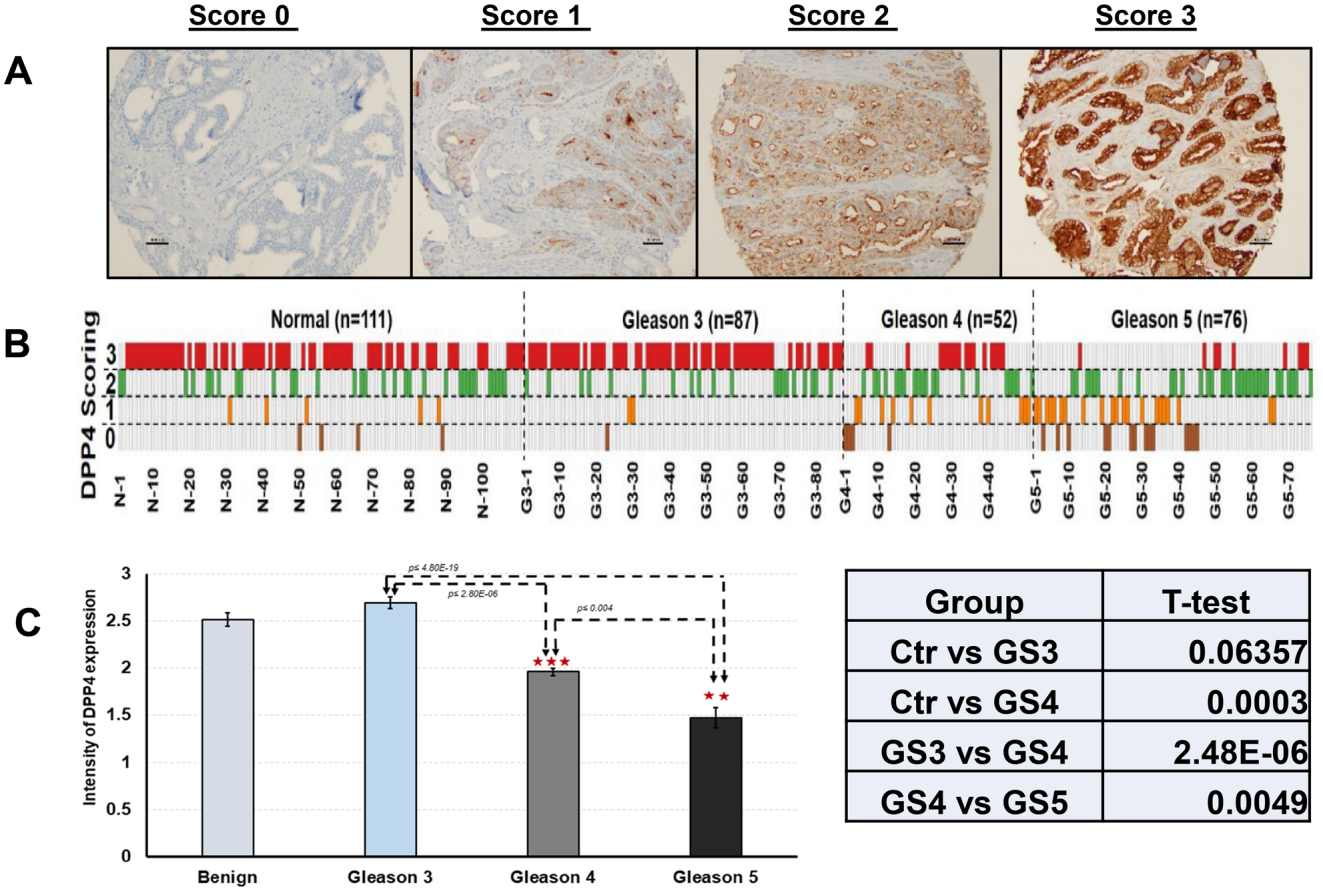
IHC staining of DPP4 expression in PCa tumor tissue microarray (TMA). (**A**) A semi-quantitative 4-tire scoring system in tumor cells: 0 (0%, no staining), 1 (< 10%, weak and focally staining), 2 (10–50%, medium and focally staining), or 3 (> 50%, strong and diffusely staining). (**B**) Correlation of DPP4 expression with Gleason scores in tumors and tumor-matched benign controls. (**C**) Comparison of DPP4 expressional pattern in tumors and tumor-matched benign controls. *P < 0.05; **P < 0.01, ***P < 0.001.

To further evaluate the expression of DPP4 in prostate tissues, IHC stain of PCa TMA was performed (Fig. 6). A total of 215 tumor cores and 111 tumor-matched benign tissue cores were constructed in the TMA. Of the tumor cores, 87 (40.5%) had a Gleason score of 3, 52 (24.2%) had a Gleason score of 4, and 76 (35.3%) had a Gleason score of 5. The staining patterns of DPP4 in tumors were analyzed using a semi-quantitative scoring system (Fig. 6A). Decreased expression of DPP4 was identified in AG tumors (Fig. 6B). A stronger staining pattern was found in Gleason score 3 tumors; in contrast, a weakly staining pattern was found in in Gleason score 4 and 5 tumors. The intensities of DPP4 staining in tumor-matched controls, Gleason 3, Gleason 4 and Gleason 5 tumors were 2.51 ± 0.75, 2.69 ± 0.57, 1.96 ± 0.92 and 1.47 ± 0.92, respectively. The DPP4 expression was significantly decreased in tumors with Gleason ≥ 4 (*P* < 0.05) (Fig. 6C).

## Discussion

PCa is a heterogeneous group of tumors. The majority of PCa is diagnosed as an indolent tumor with localized disease^8–14^, in which the patient can be cured by conventional surgical resection and/or safely observed in an active surveillance program without interventional treatments. Approximately 10% of PCa presents as lethal PCa. Recently, great efforts have been made to construct a comprehensive genomic landscape of PCa using large-scale genomic data to better understand the biology of PCa and potential therapeutic strategies. For example, the TCGA Network comprehensively characterized primary PCa and identified novel molecular features of tumors by using seven genomic platforms^3,5,9^. However, the separation of aggressive PCa from indolent tumors remains challenging. In order to gain further knowledge into the proteomic heterogeneity of aggressive PCa and investigate the molecular taxonomy of tumors for future diagnostic, prognostic, and therapeutic stratification, we profiled both primary and metastatic PCa, and comprehensively integrated those data to assess the robustness of previously defined aggressive PCa subtypes. More importantly, we also investigated the potential proteomic alterations associated with tumor progression and potential clinical indications.

One of the challenges during proteomic analysis of a relatively large number of clinical samples is to choose an effective way which can provide sufficient proteome coverage with quantitative consistency and analytic accuracy. In this study, we used the SWATH-MS platform to identify a proteomic profile of PCa. Our results demonstrated that SWATH-MS could be effectively applied for characterizing global proteomes in large-scale clinical cohorts. SWATH is an unbiased methodology that allows peptide precursor ions to be divided into several consecutive windows during fragmentation, resulting in a comprehensive fragmentation map of all detectable precursor ions for the accurate quantification of a given sample. A recent study by 11 institutions worldwide, demonstrated that SWATH-MS was a rapid, simple and reproducible method for large-scale proteomic quantitative analysis^20^. Other benefits of using SWATH-MS based technology include requiring lesser quantity of clinical samples compared to conventional DDA-based MS approaches and providing sufficient proteome coverage with quantitative consistency and analytic accuracy^15,19^.

The most unique feature of our study is the integrative analysis of different clinical stages of PCa, including PCa of Gleason score of 6 (NAG), Gleason score over 7 that resulted in death (AG), metastatic PCa with androgen deprivation therapy (M) and without androgen deprivation therapy (Nmet). In this study, a total of 4,415 proteins were identified in PCa, including 158 up-regulated and 116 down-regulated proteins in the AG subtype, comparing to NAG samples. A functional analysis demonstrated that the expression of certain proteinases such as dipeptidyl peptidase 4 (DPP4), carboxypeptidase E (CPE) and prostate specific antigen (KLK3), were significantly altered in both primary aggressive PCa and metastatic tumors compared to primary NAG PCa and normal prostate tissues. The functional role of DPP4 was further revealed by the targeted re-examination of SWATH-MS maps, including the accumulation of the DPP4 substrate, neuropeptide Y (NPY), and the reduction of NPY cleaved peptides in AG tumors.

DPP4 plays a critical role in regulating cellular signaling pathways and biological functions such as cell proliferation and migration^16,23–25^. Previously published data also suggests that the DPP4 expression is frequently lost in cancer^26,27^. By studying both primary PCa and metastatic PCa, we provide the first evidence that decreased DPP4 expression and activity is associated with PCa aggressiveness. DPP4 is an epithelial membrane-bound serine protease, which can target numerous growth factor/cytokine signaling pathways; it also has oncogenic or tumor suppressor properties^26,27^. Studies have shown that patients treated with DPP4 inhibitor, a commonly used therapy for type 2 diabetes, may accelerate PCa progression following androgen deprivation therapy^28^. A reduced serum DPP4 level was also found in PCa patients with metastatic disease^29^. Our finding of decreased DPP4 levels in aggressive and metastatic PCa is consistent with these previous studies, indicating a selective oncogenic activity to downregulate DPP4 in aggressive tumors.

The strong selective pressure to keep DPP4 levels low would then result in the accumulation of bio-active substrates. To further verify our findings, we examined the levels of enzymatic products of DPP4, including the intact substrate peptide NPY (1–36) and cleaved peptide NPY (3–36). Intact NPY (1–36) is a 36 amino acid neuropeptide that has been shown to be involved in several types of hormone-regulated cancer, including breast, ovarian and prostate cancer^30–32^, where it modulates tumor cell proliferation through the activation of the Y1-receptor signaling pathway^32–34^. Interestingly, cleaved NPY (3–36) loses its ability to activate the Y1-receptor, and becomes a selective activator of the Y2-receptor^35^. These studies suggested that NPY (1–36) and NPY (3–36) may initiate different intracellular signaling pathways. The decreased DPP4 activity in aggressive PCa may lead to increased tumor cell proliferation through the accumulation of the substrate peptides NPY (1–36). Taken together, our findings suggest that loss of DPP4 expression and activity may promote prostate cancer aggressiveness through the regulatory effect of NPY (1–36). Decreased expression of DPP4 and the subsequent increase in bio-active substrate levels promote tumor cell proliferation and disease progression. It can serve as a signature of AG tumors.

Recently several proteomic studies have published to comprehensively proteomic characterization of PCa, including profiling potential molecular mechanisms and comparing proteomics with genomic and epigenetic findings in the progression of PCa^36–42^. In the study of 28 primary PCa (Gleason score 6–9), authors found that an increased expression of pro-NPY was associated with a poor prognosis^36^. Sinha A, et al. studied the proteomic signatures of 76 localized, intermediate-risk PCa tumor tissues, and identified four distinct protein clusters correlated with five clinical groups^37^. They also found that proteomic signature was also correlated with genomic subtypes of PCa. Interestingly, they found that changes of mRNA abundance could not reflex the protein abundance variability. Their findings indicated the importance of multiomic proteogenomic study of PCa^37^. In a comparative study of PCa cell lines with PCa tumor tissues, 12 mutant peptides were identified to be differentially expressed in PCa tumor tissue^38^. Similarly, several proteins were also identified to be differentially expressed in subtypes of PCa^39–42^. Indeed, the dysregulation of protein levels is also mirrored by alterations of specific genes^43^. All these proteogenomic findings demonstrate that extensive intracellular signaling pathways are involved in PCa. A multi-modal proteomic analysis is necessary for the identification of potential clinical protein biomarkers^44^.

In summary, we analyzed PCa tumor tissues, including primary NAG, AG, and metastatic tumors, using a DIA SWATH–MS platform. We identified a comprehensive proteomic map containing 4,415 proteins. We also characterized AG-associated proteins, including 158 up-regulated and 116 down-regulated proteins. A functional analysis of tumor-associated proteins revealed the reduced expression of several proteinases, including dipeptidyl peptidase 4 (DPP4), carboxypeptidase E (CPE) and prostate specific antigen (KLK3), particularly in AG and metastatic PCa. We identified accumulation of the DPP4 substrate, neuropeptide Y (NPY), and the reduction of NPY cleaved peptides in AG tumors by the targeted re-examination of SWATH-MS maps. The decreased level of DPP4 in AG was further validated using an independently-collected cohort, as well as by comparison with a TCGA mRNA dataset and the immunohistochemical stains using our tumor microarray (TMA). Our findings demonstrate that DPP4 plays a critical role in PCa progression. It may serve as a clinical biomarker. Additional studies are necessary to determine the clinical significance of these proteinases and their potential diagnostic and therapeutic value.

## Methods and materials

### Materials

BCA protein assay kit, Urea, and tris (2-carboxyethyl) phosphine (TCEP) were from Thermo Fisher Scientific (Waltham, MA); sequencing-grade trypsin was from Promega (Madison, WI); C18 columns and Strong Cation Exchange (SCX) columns were from Glygen (Columbia, MD); all other chemicals were from Sigma-Aldrich (St. Louis, MO).

### Clinical samples collection and preparation

Samples were collected from the Project to Eliminate Lethal Prostate Cancer (PELICAN) rapid autopsy program at the Johns Hopkins Autopsy Study of Lethal Prostate Cancer (JHASPC) initiated in 1994. Tumor samples were obtained from radical prostatectomy, transurethral resection and/or autopsy. Benign control samples were obtained from transplant patients in autopsy service, who did not have history of PCa. All samples collected with patients’ informed consents and in a manner to protect patients’ identity. A total of 106 samples were included in the study, including 48 cases of primary and 48 cases of metastatic PCa, as well as 10 benign prostate controls. Among the samples, the discovery cohort included 10 NAG, 9 AG and 20 metastatic PCa, whereas, the independently-collected validation cohort included 19 NAG, 10 AG and 28 metastatic PCa. All specimens were snap-frozen, embedded in optimal cutting temperature (OCT) compound and stored at − 80 °C until use. OCT-embedded frozen tumor tissues were sectioned and enriched using a cryostat microdissection as previously described^16,45^. Proteins were extracted using 8 M urea and digested with trypsin^46,47^. Digested peptides were thoroughly cleaned with C18 and SCX columns, vacuum dried and resuspended in 0.2% formic acid.

The hematoxylin and eosin (H&E) stained tumor sections were reviewed by pathologists to ensure the representation of tumor area. The study was approved by the Institutional Review Board of Johns Hopkins Medical Institutions. In addition, all methods used for this study were performed in accordance with the relevant guidelines and regulations.

### Data dependent (DDA) LC–MS/MS on 5600^+^ Triple TOF

To build the spectral library, an equal amount of peptides from each tissue group in the discovery cohort were pooled, and then, the pooled samples were separated to 24 fractions with high pH reversed-phase chromatography as previously described ^48^. Each fraction was analyzed on a SCIEX 5600^+^ Triple TOF mass spectrometer (SCIEX, Framingham, MA) with an Eksigent ekspertTM nanoLC 400 system in DDA mode. Peptides were loaded onto a 200 µm × 0.5 mm cHiPLC trap column followed by a 75 µm × 15 cm nano cHiPLC column, and separated using a 90-min gradient from 5 to 35%, buffer A 0.1% (v/v) formic acid in water, buffer B 0.1% (v/v) formic acid in ACN at a flow rate of 300 µL/min. The MS1 spectra were collected in the range 400–1,800 m/z for 250 ms. The 30 most intense precursors with a charge state of 2–5 which exceeded 50 counts per second were selected. The MS2 spectra was collected in the range 100–1,800 m/z for 50 ms. Precursor ions were dynamically excluded from reselection for 6 s. The duty cycle time was ~ 1.8 s.

### Protein identification and quantification

MS/MS spectra of 24 fractionated raw data from the 5600^+^ TripleTOF were searched using ProteinPilot 4.5 against the UniProtKB/Swiss-Prot complete human proteome database containing 20,274 entries (version of December, 2013). The database search included static modifications of 57.021 Da for cysteine, dynamic modification of 15.995 Da for oxidation, and dynamic modification of 42.011 Da for acetylation (N-terminus only). Ion libraries were generated using PeakView from ProteinPilot search files for proteins identified at 1% FDR. MS/MS intensities of selected transitions of non-shared peptides were extracted and quantified using PeakView 2.0 with the SWATH all MS/MS 2.0 microapp. Protein identification was counted based on all the peptides passing 1% FDR requirement in individual sample. Protein quantification was the sum of abundances of all corresponding peptides passing 1% FDR requirement in at least one sample.

### SWATH-MS measurement

SWATH-MS datasets from 106 tissue samples were acquired using a 5600^+^ Triple TOF mass spectrometer. The chromatographic system and settings were the same as those for DDA LC–MS/MS as described above. In SWATH-MS mode, the instrument was optimized to quadrupole settings for the selection of 25-m/z wide precursor ion selection windows. Using an isolation width of 26 m/z (containing 1 m/z for the window overlap), 34 overlapping windows were constructed covering the precursor mass range of 400–1,250 m/z. SWATH MS2 spectra were collected from 100 to 1,800 m/z. The collision energy (CE) was optimized for each window according to the calculation for a charge 2 + ion centered upon the window with a spread of 15 eV. An accumulation time (dwell time) of 100 ms was used for all fragment-ion scans in high-sensitivity mode. For each SWATH-MS cycle, a survey scan in high-resolution mode was also acquired for 50 ms, resulting in a duty cycle of ~ 3.5 s.

### Bioinformatics analysis of SWATH proteomics data

All samples were normalized to the reference (aggressive PCa sample number 1 (AG1)) based on the assumption that all samples have a median relative expression of 1. The absolute protein abundances were transformed to log2 ratio values. The protein biological coefficient of variation (CV) was calculated based on the CV of the log2 averaged abundances of all samples. The protein technical CVs were calculated based upon replicates of samples in 5 tissue groups (C, NAG, AG, Nmet and M); the maximum value from these groups was used. Proteins with a biological CV less than the technical CV were not used for the further analysis. 3865 out of 4415 proteins were included in further analysis.

Unsupervised hierarchical clustering was performed using the clustermap function of the Seaborn Python package (metric = ‘euclidean’, and method = ‘ward’)^49^. Proteins with the highest variances (the top 500 proteins) were included in the clustering and annotated for pathway enrichment analysis. Functional analysis such as Over-Representation Analysis (ORA) of differentially expressed proteins between sample clusters was performed using WebGestalt (http://www.webgestalt.org/)^50^. Protein features were evaluated against a background dataset of 3865 proteins quantified by the SWATH-MS analysis. Principal component analysis (PCA) was performed by OmicsOne^51^ to further investigate the sample distances between the cancer subtypes and control samples.

Differential analysis was performed on all qualified proteins passed CV filtration (including tumors verse control as well as AG verse NAG). For tumors and control comparison, the student’s t-test p-values were adjusted by the Benjamin-Hochberg (BH) correction. The significantly up- and down-regulated proteins were labelled if adjusted p values < 0.01 and absolute fold changes ≥ 2 during the t-tests. For AG and NAG comparison due to the limited number of samples, proteins with ≥ 1.5 fold changes and a p value < 0.05 were considered to be subtype-associated proteins. TCGA RNA-seq data was downloaded from the Genomic Data Commons Data Portal (https://portal.gdc.cancer.gov/) for the verification study.

A permutation test was performed to select subtype-associated stable proteins discriminating between AG and NAG primary tumors. In the test, protein expression data from AG and NAG groups were generated by random sampling (Permutated). The procedure was performed 500 times. Proteins were ranked by p values with the smallest p value having rank 1, based upon student’s t-test on log-transformed data. The average rank was plotted for permutated and non-permutated analysis against the number of proteins.

The log2 fold changes of proteins were analyzed by the Gene Set Enrichment Analysis (GSEA) function of WebGestalt to find the enriched Gene Ontology (GO) terms of biological process and molecular function databases under 5% FDR.

### Immunohistochemistry of PCa tumor tissue microarray

The PCa tissue microarray (TMA) was constructed using surgical resected tumors (n = 60 cases), including Gleason score 3 (i.e. 3 + 3), 4 (i.e. 3 + 4, 4 + 3, or 4 + 4) and 5 (i.e. 5 + 4 or 4 + 5) tumors. All tissues were fixed in 10% buffered formalin and embedded in paraffin. The 6 mm core was used for the TMA, including 215 cores of PCa and 111 cores of adjacent tumor-matched benign tissue. The use of human tumor tissue was approved by the Johns Hopkins Institutional Review Board.

Immunohistochemical (IHC) study of DPP4 was performed on TMA using a Ventana Discovery Ultra autostainer (Roche Diagnostics). Briefly, following dewaxing and rehydration on board, epitope retrieval was performed using Ventana Ultra CC1 buffer (catalog# 6,414,575,001, Roche Diagnostics) at 96 °C for 48 min. Primary antibody, anti-DD4/CD26 (D6D8K, catalog #67,138, Cell signaling Inc) at 1:250 dilution was applied at 36 °C for 60 min. Primary antibodies were detected using an anti-rabbit HQ detection system (catalog# 7,017,936,001 and 7,017,812,001, Roche Diagnostics) followed by a Chromomap DAB IHC detection kit (catalog # 5,266,645,001, Roche Diagnostics), counterstaining with Mayer’s hematoxylin, dehydration and mounting. The intensity of the IHC staining pattern was semi-quantitatively by two researchers QKL (the American Board of Pathology certified pathologist) and NH, using a 4-tier system, which was as follows: 0 (0%, no staining), 1 (< 10%, weak and focally staining), 2 (10–50%, medium and focally staining), or 3 (> 50%, strong and diffusely staining) in tumor cells.

### Consent for publication

This manuscript has been read and approved by all the authors to publish and is not submitted or under consideration for publication elsewhere. This manuscript has been read and approved by all the authors to publish and is not submitted or under consideration for publication elsewhere.

## Supplementary Information

Supplementary Information 1.

Supplementary Information 2.

Supplementary Information 3.

Supplementary Information 4.

Supplementary Information 5.

Supplementary Information 6.

## Abbreviations

PCa: Prostate cancer
AG: Aggressive prostate cancer
NAG: Non-aggressive prostate cancer
C: Control
M: Metastasis with androgen deprivation therapy
Nmet: Metastasis without androgen deprivation therapy
USPSTF: US preventive services task force
ISUP: International society of urological pathology
MS: Mass spectrometry
TMA: Tumor microarray
DIA: Data-independent acquisition
SWATH: The sequential window acquisition of all theoretical fragment ion spectra
TCGA: The Cancer Genome Atlas
DDA: Data-dependent acquisition
TCEP: Tris (2-carboxyethyl) phosphine
SCX: Strong cation exchange column
OCT: Optimal cutting temperature
H&E: Hematoxylin and eosin
ACN: Acetonitrile
FDR: False discovery rate
CE: Collision energy
CV: Coefficient of variations
PCA: Principal component analysis
BH: Benjamin-Hochberg
SD: Standard deviation
HPA: Human Proteome Atlas
QC: Quality control
GO: Gene Ontology
GSEA: Gene set enrichment analysis
FDA: Food and Drug Administration
IHC: Immunohistochemistry
PELICAN: Project to eliminate lethal prostate cancer
JHASPC: The Johns Hopkins autopsy study of lethal prostate cancer
ORA: Over-representation analysis
DPP4: Dipeptidyl peptidase 4
KLK3: Prostate specific antigen
CMA1: Chymase 1
CPE: Carboxypeptidase E
ITGB1: Integrin beta-1
ANPEP: Aminopeptidase N
NPY: Neuropeptide Y
