# Supplementary material for: Proteomic characterization of primary and metastatic prostate cancer reveals reduced proteinase activity in aggressive tumors
Source: Sci Rep. 2021 Sep 23;11:18936. doi: 10.1038/s41598-021-98410-0 (PMC8460832; doi:10.1038/s41598-021-98410-0)
Supplement: Supplementary file 1 — Supplementary Information 1. [file 41598_2021_98410_MOESM1_ESM.docx]

**Supplementary data**

**Supplementary Table 1**. Clinical information.

**Supplementary Table 2**. Protein identifications and counting.

**Supplementary Table 3**. Protein clustering and gene set enrichment.

**Supplementary Table 4**. Differential analysis on NAG and AG groups.

**Supplementary table 5**. TCGA clinical information.
